# Relativistic Prolapse-Free Gaussian Basis Sets of Double- and Triple‑*ζ* Quality for f‑Block Elements: (aug-)RPF-2Z and (aug-)RPF-3Z

**DOI:** 10.1021/acs.jctc.6c00601

**Published:** 2026-09-08

**Authors:** Anne Kéllen de Nazaré dos Reis Dias, Julielson dos Santos Sousa, Eriosvaldo Florentino Gusmão, Roberto Luiz Andrade Haiduke

**Affiliations:** Department of Chemistry and Molecular Physics, São Carlos Institute of Chemistry, 28133 University of São Paulo, São Carlos, São Paulo 13566-590, Brazil

## Abstract

This study presents the relativistic prolapse-free Gaussian basis sets of double- and triple-*ζ* quality (RPF-2Z and RPF-3Z, respectively) developed for *f*-block elements. Thus, RPF-2Z and RPF-3Z are achieved after the augmentation of small- and medium-size sets of primitive functions previously generated, respectively, by correlation/polarization (C/P) functions obtained by relying on the power of a polynomial version of the Generator-Coordinate Dirac-Fock method aiming a description of the valence space. These C/P functions are selected based on the results of multi-reference configuration interaction calculations and also considering consistency arguments when compared to the already proposed RPF-4Z set for the same elements. Versions of these new sets containing extra diffuse functions (aug-RPF-2Z and aug-RPF-3Z) are promptly defined as well. Calculations of fundamental molecular properties show that these new basis sets constitute reliable alternatives to well-known sets of similar quality. Finally, due to the absence of variational prolapse, the new sets must show advantages in investigations of properties that are more strongly affected by the distribution of the innermost electrons.

## Graphical abstract

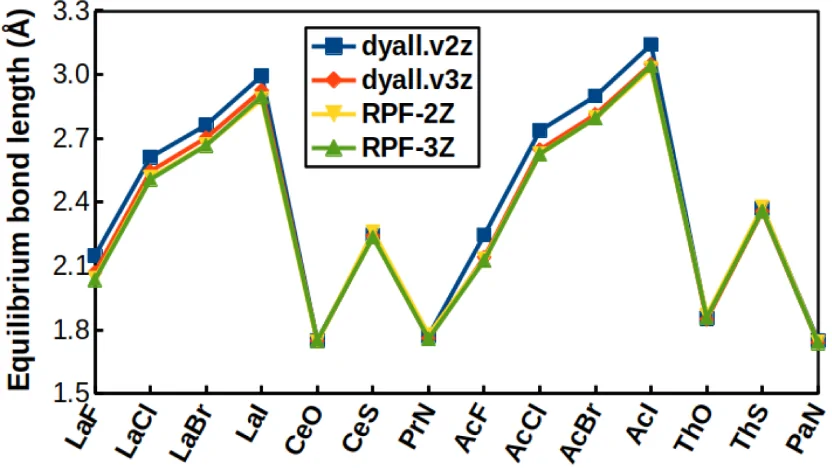

## 1 Introduction

The relativistic effects on electronic structure are actually included by means of the four-component treatment, firmly established by Paul Dirac in 1928,^1^ or by means of more approximate alternatives such as two-component transformation methods, which are chosen in order to achieve computational demand savings. For instance, a modern two-component implementation^2^ is actually known as the exact two-component (X2C) formalism. In addition, X2C can also be found in a variant that excludes spin-orbit coupling effects,^3^ named spin-free X2C (X2C-SF). These methods are among the best workhorses available today for including relativistic effects in quantum chemistry, with widespread practical applications in more advanced investigations on atomic and molecular properties. In all such cases, it is fundamental to use reliable and efficient basis sets. Thus, although non-relativistic sets have been considered in early four-component calculations,^4^ the development of new alternatives is a relevant research subject in this context.

However, a basis-set incompleteness deficiency named variational prolapse by Faegri^5^ appears as a result of neglecting the minimax theorem of Talman^6^ along the exponent optimization procedure and is associated with deficiencies in spinor descriptions in the innermost atomic regions.^4^ As a consequence, basis sets plagued by this issue can provide electronic energies below the exact results, although the effect of prolapse is not so brutal as the one observed in early calculations with variational collapse problems, which were solved by the implementation of kinetic-balance conditions between large and small components basis-set constituents.^7,8^ In this context, the polynomial version of the generator coordinate Dirac-Fock (p-GCDF) method^9^ can produce compact sets free of any variational prolapse signs, namely the small- and medium-size relativistic prolapse-free Gaussian basis sets (SRPF and MRPF, respectively).^10–12^

Similar strategies were used before in our research group for generating larger primitive sets, which, after being augmented by correlation/polarization (C/P) functions aiming at a better description of the valence space, provided relativistic prolapse-free Gaussian basis sets of quadruple-*ζ* quality, RPF-4Z.^13–15^ Hence, RPF-4Z is available for all 118 known elements of the Periodic Table, providing a computationally efficient alternative to popular sets of similar size. Of course, SRPF and MRPF sets can also be used as starting points for reaching relativistic prolapse-free Gaussian basis sets of double- and triple-*ζ* quality (RPF-2Z and RPF-3Z, respectively). This step was initiated by simpler *s*- and *p*-block elements, a work finished in 2024,^16^ and extended soon later to *d*-block atoms.^17^ Readers more interested in the development of relativistic basis sets are referred to these previous papers.

Now, this investigation intends to conclude the task, focusing on *f*-block elements (lanthanides and actinides). Again, RPF-2Z and RPF-3Z are also accompanied by versions of these sets including extra diffuse functions for all of the function symmetries available (*s*, *p*, *d*, *f*, *g*, and *h*), reaching the aug-RPF-2Z and aug-RPF-3Z sets, respectively. Calculations of fundamental molecular properties in diatomic systems are also conducted here to evaluate the comparative performance of RPF-2Z and RPF-3Z. Thus, since the new sets are free of variational prolapse, they should exhibit advantages being also recommended for applications in challenging properties for other relativistic sets available today, such as innermost electron excitation processes.^4^ In fact, the RPF-4Z sets are being used to obtain quite accurate electric field gradient (EFG) values at nuclei of interest, providing reliable data of nuclear quadrupole moments (NQMs), as done recently for barium.^18^

## 2 Methodology

The p-GCDF method^9^ truncated at third order provides Gaussian function exponents $\left(\gamma_{i}^{\left(w\right)}\right)$ by means of the following relations:

1 $$\Theta_{i}^{\left(w\right)}=\frac{\ln\gamma_{i}^{\left(w\right)}}{\alpha }=\Theta_{min}^{\left(w\right)}+\overset{3}{\underset{q=1}{\sum }}\Delta \Theta_{q}^{\left(w\right)}{\left(i-1\right)}^{q}$$

and

2 $$\gamma_{i}^{\left(w\right)}=\exp\left\{\alpha \left[\Theta_{min}^{\left(w\right)}+\overset{3}{\underset{q=1}{\sum }}\Delta \Theta_{q}^{\left(w\right)}{\left(i-1\right)}^{q}\right]\right\}$$

where *w* denotes the function symmetry (*s*, *p*, *d*, *f*, and so on). Moreover, *i* = 1, 2, ..., *N* (*N* labels the number of discretization points), *α* is a scaling parameter that is set as 6.0,^19^ while $\Theta_{\min}^{\left(w\right)}$ and $\Delta \Theta_{q}^{\left(w\right)}$ are parameters corresponding, respectively, to the mesh initial point and the increment of order *q* for providing the discretization points.

Here, this same method, which was initially used for generating the starting primitive Gaussian sets (SRPF and MRPF),^10–12^ was now employed to obtain the C/P function exponents. These C/P Gaussian functions can be more diffuse than the ones already found for a certain *w* symmetry in the SRPF and MRPF sets (obtained by extrapolations done using *i* ≤ 0), or they can be functions of higher angular-momentum symmetries than those available in the SRPF or MRPF sets, which are achieved by using the p-GCDF parameters of the *w* – 2 angular symmetry.^13–15^ In more detail, non-null positive integer *i* values are used to provide SRPF and MRPF sets, while *i* = 0, −1, −2, and so on, readily result in Gaussian exponents that are associated with more and more diffuse functions. Finally, the choice made for achieving higher angular-momentum functions aims to reduce the number of small-component functions resulting from the kinetic-balance conditions, which should decrease the demands for computational resources. This strategy is similar to the idea behind dual-family basis sets.^5^

## 3 Computational Details

The electronic-structure calculations were done here by means of the DIRAC23 package.^20,21^ The default Gaussian nuclear model^22^ was always selected along with the standard light speed value of 137.0359992 atomic units (*au*). In order to reduce the demands for computational resources, two-electron integrals involving only small-component functions (SS|SS) were replaced by a simple Coulombic approximation.^23^ The basis sets were always kept uncontracted. Moreover, the standard kinetic-balance conditions (upward and downward) were used for generating small-component functions from the large-component ones.^7,8^ Hence, only large-component functions are discussed in this paper.

The multi-reference Configuration Interaction calculations with single and double excitations (MR-CISD), which are based on Dirac-Fock-Coulomb (DFC) wavefunctions, are done for selecting the C/P functions. In this case, the direct relativistic configuration interaction (DIRRCI) module^24^ of DIRAC23 is called to perform this task. Moreover, the Davidson correction within MR-CISD was included whenever available. The Restricted Active Space (RAS) strategy was chosen here. First, RAS1 was not defined, implying that deep-core and sub-valence spinors are kept frozen. Thus, the *ns* electrons (6*s* or 7*s* for lanthanides and actinides, respectively), along with (*n* – 2)*f* and (*n* – 1)*d* electrons within the experimental ground-state configuration of each element and the respective spinors, are included into the RAS2 valence space. This means that 3–16 active electrons are considered as one moves along these sub-blocks. In other words, all possible occupation schemes of valence electrons and spinors are possible. Finally, RAS3 is generally constituted by other virtual spinors with energy up to 20.0 *au*, although some calculations required increasing this value up to 75.0 *au* (investigations of *g* and *h* functions for RPF-3Z in lanthanides). Moreover, due to high demands for computational resources of many of these MR-CISD calculations, the C/P selection procedure is done individually, one symmetry at a time (*s*, *p*, *d*, *f*, *g*, and *h*). For instance, the analysis of *f* functions is done without including any of the previously selected functions of other angular momenta. An exception occurs as one considers *h* functions, where these calculations are done based on a basis set also containing the best *g* function. Moreover, *g* and *h* functions for lanthanum are tested in terms of a basis set already including *f* functions.

Molecular calculations were performed to carry out an evaluation of the new basis sets. In this context, the results were compared to those from popular alternative basis sets of similar quality, dyall.v2z and dyall.v3z.^25,26^ However, because of persistent convergence errors detected at this stage, the X2C-SF approach is now used. Thus, the coupled-cluster method with iterative single and double excitations, along with a perturbative correction for triple substitutions^27,28^ is employed to include electron correlation effects on these molecular investigations (X2C-SF-CCSD-T). The active space encompasses 18 or 28 electrons (28 electrons are only taken into account for molecules with iodine) and virtual spinors with energies up to 10.0 *au*, with a gap of 1.0 *au*. The following diatomic molecules were considered in their lowest-lying singlet spin state: LaF, LaCl, LaBr, LaI, CeO, CeS, PrN, AcF, AcCl, AcBr, AcI, ThO, ThS, and PaN. This choice is needed due to the computational limitations of the current implementation within DIRAC23, although this state may not be the ground state for some of these systems. In fact, the experimental ground state is not known for some of these molecules, but lanthanum halides, ThO, and ThS are certainly closed-shell systems.

Equilibrium bond lengths (*r*_*e*_) and harmonic vibrational frequencies (*w*_*e*_) are achieved through a fourth-order polynomial fit of the potential energy curve (PEC) constructed from seven X2C-SF-CCSD-T energy points from geometries with bond lengths equally displaced by 0.01 Å, which are sampled around the energy minimum obtained with each basis set. The PEC was fitted through the TWOFIT utility program within the DIRAC package by using the atomic masses of the most abundant isotopes, unless explicitly mentioned. Molecular dipole moments are determined at the equilibrium geometries of each basis set (*μ*_*e*_) through the sum of the analytical SF-DFC expectation value with X2C-SF-CCSD-T electron correlation contributions given by the finite-difference technique in a two-point form, described by

3 $${\left(\frac{\partial E\left(\lambda \right)}{\partial \lambda }\right)}_{0}\approx -\left(\frac{E\left(+\lambda \right)-E\left(\lambda \right)}{2\lambda }\right)$$

where *E* is the electron correlation energy and λ is the strength of the perturbation inserted into the Hamiltonian, taken as 1×10^–6^ *au*.

## 4 Basis-Set Augmentation with Correlation/Polarization Functions

### 4.1 RPF-2Z and RPF-3Z Sets

The number and type of C/P functions chosen for composing the RPF-2Z and RPF-3Z sets must also be consistent with the previously developed quadruple-*ζ* quality set of this family (RPF-4Z) for lanthanides and actinides.^15^ The starting primitive sets of these elements (SRPF and MRPF)^11,12^ already contain functions of *s*, *p*, *d*, and *f* symmetries (except for La, which also lacks *f* functions in its ground-state configuration). Thus, for a more uniform treatment of all elements, we decided to maintain the basis-set size constant along each sub-block, as done before.^15^ This is a relevant rule once the primitive sets present different sizes depending on the ground-state configuration of each element. Thus, more diffuse functions of *s*, *p*, *d*, and *f* symmetries are initially evaluated here.

The RPF-4Z of the lanthanides includes [1*s*2*p*(1,3)*d*1*f*] functions in the C/P set, although La needs 12 *f* functions to achieve the same basis set size of the other elements within this sub-block.^15^ The number of *d* functions inserted (1 or 3) varies for similar reasons. Thus, for consistency, RPF-2Z and RPF-3Z follow the same pattern, that is, starting with [1*s*2*p*(1,3)*d*1*f*] additional functions, except again for La. In the case of lanthanum, considering the fixed-size argument along sub-blocks, 10 and 11 *f* functions are chosen for RPF-2Z and RPF-3Z, respectively. Again, the ground-state electron configurations containing occupied 5*d* orbitals, [Xe]­4*f* ^*Z*–57^5*d* ^1^6*s* ^2^, receive a single extra *d* function,[Fn fn1] while the others, [Xe]­4*f* ^*Z*–56^6*s* ^2^, require three *d* functions to achieve the same basis set size.

Next, the RPF-4Z set of actinides encompasses [2*s*2*p*(1,3)*d*(1,4)*f*] functions into the C/P set.^15^ In analogy to the previous discussion in terms of constant basis set size arguments along the sub-block, [2*s*2*p*(1,3)*d*(1,3)*f*] C/P functions are inserted into the RPF-3Z set, while RPF-2Z requires [2*s*2*p*(1,2)*d*(1,2)*f*] C/P functions. In more detail, electron configurations such as [Rn]­5*f* ^*Z*–89^6*d* ^1^7*s* ^2^ and the one seen for Th receive one *d* function, while the remaining ones, [Rn]­5*f* ^*Z*–88^7*s* ^2^, need more *d* functions (2 or 3 for RPF-2Z and RPF-3Z, respectively). The two initial actinides that still do not present filled 5*f* orbitals, Ac and Th, also require more *f* functions (2 or 3 for RPF-2Z and RPF-3Z, respectively) than the others, which are augmented by a single *f* function.

Next, functions of larger angular momentum are also addressed to complement the C/P group. In this context, RPF-4Z includes functions with angular momentum up to *i* for these *f*-block elements.^15^ Thus, considering the basis set hierarchy within the family, it seems reasonable to consider one *h* plus a few *g* functions for a triple-*ζ* set (RPF-3Z) and a minimum augmentation by a single *g* function for the smallest set, RPF-2Z. As mentioned earlier, *g* and *h* functions share the same exponents of *d* and *f* functions already incorporated, respectively, and the *i* indices in eq 2 are defined by the MR-CISD calculations, except for Gd and Cm. In these two cases, the MR-CISD calculations are unfeasible with our computational resources, and the choice was guided by an exponent increase pattern with the atomic number noticed for the other elements.

Hence, the best *g* function for RPF-2Z was chosen by means of MR-CISD calculations, which pointed out the particular function capable of causing the largest energy reduction. Next, we observed that the optimal subset of higher angular momentum C/P functions for the RPF-3Z set of the *f*-block elements corresponds to the inclusion of [3*g*,1*h*] functions. The three *g* functions selected here are the ones that resulted in the most relevant energy decreases when considered individually. This choice for [3*g*1*h*] additional C/P functions of larger angular momentum seems to be a better option because the energetic effect of the first *h* function is similar to that obtained during the addition of a third *g* function for the majority of these elements. Representative examples are displayed in Table 1. In other words, this selection is recommended, considering correlation consistency arguments.

**Table 1:** Energetic Effect of *G* and *H* Functions Obtained in MR-CISD Calculations Done to Achieve the RPF-3Z Set of a Few Selected Elements (in mHartree)

Element	First *g*	Second *g*	Third *g*	First *h*
_57_La	–0.64	–0.15	–0.09	–0.11
_70_Yb	–148.31	–86.82	–13.53	–26.56
_89_Ac	–1.35	–0.17	–0.10	–0.04
_102_No	–225.00	–77.87	–32.99	–40.06

Finally, Tables 2 [Table tbl3] [Table tbl4],5, display the *i* indices that define the chosen C/P functions according to eq 2. As one can see, the elements with electron configurations in accordance with the Aufbau principle, [Xe]­4*f* ^*Z*–56^6*s* ^2^ or [Rn]­5*f* ^*Z*–88^7*s* ^2^, present *i* values that tend to be maintained or increase in line with the atomic number, a well-behaved pattern noticed before as the p-GCDF method was used for this purpose.^15^ A similar trend is also observed among the group of elements with alternative electron configurations, [Xe]­4*f* ^*Z*–57^5*d* ^1^6*s* ^2^ or [Rn]­5*f* ^*Z*–89^6*d* ^1^7*s* ^2^.

**Table 2:** Selected Indexes for the Correlation/Polarization Functions of the RPF-2Z Set for Lanthanides

Element	electronic configuration	*s*	*p*	*d*	*f*	*g*
_57_La	[Xe]5*d* ^1^6*s* ^2^	0	–1,0	0	0,···,9	2
_58_Ce	[Xe]4*f* ^1^5*d* ^1^6*s* ^2^	0	–1,0	0	0	3
_59_Pr	[Xe]4*f* ^3^6*s* ^2^	0	–1,0	–2,-1,0	0	2
_60_Nd	[Xe]4*f* ^4^6*s* ^2^	0	–1,0	–2,-1,0	0	2
_61_Pm	[Xe]4*f* ^5^6*s* ^2^	0	–1,0	–2,-1,0	0	2
_62_Sm	[Xe]4*f* ^6^6*s* ^2^	0	–1,0	–2,-1,0	0	3
_63_Eu	[Xe]4*f* ^7^6*s* ^2^	0	–1,0	–2,-1,0	0	3
_64_Gd	[Xe]4*f* ^7^5*d* ^1^6*s* ^2^	0	–1,0	0	0	5
_65_Tb	[Xe]4*f* ^9^6*s* ^2^	0	–1,0	–2,-1,0	0	3
_66_Dy	[Xe]4*f* ^10^6*s* ^2^	0	–1,0	–2,-1,0	0	3
_67_Ho	[Xe]4*f* ^11^6*s* ^2^	0	–1,0	–2,-1,0	0	3
_68_Er	[Xe]4*f* ^12^6*s* ^2^	0	–1,0	–2,-1,0	0	3
_69_Tm	[Xe]4*f* ^13^6*s* ^2^	0	–1,0	–2,-1,0	0	3
_70_Yb	[Xe]4*f* ^14^ 6*s* ^2^	0	–1,0	–2,-1,0	0	3

**Table 3:** Selected Indexes for the Correlation/Polarization Functions of the RPF-2Z Set for Actinides

Element	electronic configuration	*s*	*p*	*d*	*f*	*g*
_89_Ac	[Rn]6*d* ^1^7*s* ^2^	–1,0	–1,0	0	–1,0	2
_90_Th	[Rn]6*d* ^2^7*s* ^2^	–1,0	–1,0	0	–1,0	2
_91_Pa	[Rn]5*f* ^2^6*d* ^1^7*s* ^2^	–1,0	–1,0	0	0	3
_92_U	[Rn]5*f* ^3^6*d* ^1^7*s* ^2^	–1,0	–1,0	0	0	3
_93_Np	[Rn]5*f* ^4^6*d* ^1^7*s* ^2^	–1,0	–1,0	0	0	4
_94_Pu	[Rn]5*f* ^6^7*s* ^2^	–1,0	–1,0	–1,0	0	2
_95_Am	[Rn]5*f* ^7^7*s* ^2^	–1,0	–1,0	–1,0	0	2
_96_Cm	[Rn]5*f* ^7^6*d* ^1^7*s* ^2^	–1,0	–1,0	0	0	4
_97_Bk	[Rn]5*f* ^9^7*s* ^2^	–1,0	–1,0	–1,0	0	2
_98_Cf	[Rn]5*f* ^10^7*s* ^2^	–1,0	–1,0	–1,0	0	2
_99_Es	[Rn]5*f* ^11^7*s* ^2^	–1,0	–1,0	–1,0	0	2
_100_Fm	[Rn]5*f* ^12^7*s* ^2^	–1,0	–1,0	–1,0	0	2
_101_Md	[Rn]5*f* ^13^7*s* ^2^	–1,0	–1,0	–1,0	0	2
_102_No	[Rn]5*f* ^14^7*s* ^2^	–1,0	–1,0	–1,0	0	2

**Table 4:** Selected Indexes for the Correlation/Polarization Functions of the RPF-3Z Set for Lanthanides

Elem.	electronic configuration	*s*	*p*	*d*	*f*	*g*	*h*
_57_La	[Xe]5*d* ^1^6*s* ^2^	0	–1,0	0	0,···,10	1,2,3	1
_58_Ce	[Xe]4*f* ^1^5*d* ^1^6*s* ^2^	0	–1,0	0	0	2,3,4	2
_59_Pr	[Xe]4*f* ^3^6*s* ^2^	0	–1,0	–2,-1,0	0	1,2,3	5
_60_Nd	[Xe]4*f* ^4^6*s* ^2^	0	–1,0	–2,-1,0	0	1,2,3	5
_61_Pm	[Xe]4*f* ^5^6*s* ^2^	0	–1,0	–2,-1,0	0	2,3,4	5
_62_Sm	[Xe]4*f* ^6^6*s* ^2^	0	–1,0	–2,-1,0	0	2,3,4	5
_63_Eu	[Xe]4*f* ^7^6*s* ^2^	0	–1,0	–2,-1,0	0	2,3,4	5
_64_Gd	[Xe]4*f* ^7^5*d* ^1^ 6*s* ^2^	0	–1,0	0	0	4,5,6	5
_65_Tb	[Xe]4*f* ^9^6*s* ^2^	0	–1,0	–2,-1,0	0	2,3,4	5
_66_Dy	[Xe]4*f* ^10^6*s* ^2^	0	–1,0	–2,-1,0	0	2,3,4	5
_67_Ho	[Xe]4*f* ^11^6*s* ^2^	0	–1,0	–2,-1,0	0	2,3,4	5
_68_Er	[Xe]4*f* ^12^6*s* ^2^	0	–1,0	–2,-1,0	0	2,3,4	5
_69_Tm	[Xe]4*f* ^13^6*s* ^2^	0	–1,0	–2,-1,0	0	2,3,4	5
_70_Yb	[Xe]4*f* ^14^6*s* ^2^	0	–1,0	–2,-1,0	0	2,3,4	5

**Table 5:** Selected Indexes for the Correlation/Polarization Functions of the RPF-3Z Set for Actinides

Elem.	electronic configuration	*s*	*p*	*d*	*f*	*g*	*h*
_89_Ac	[Rn]6*d* ^1^7*s* ^2^	–1,0	–1,0	0	–2,-1,0	1,2,3	–1
_90_Th	[Rn]6*d* ^2^7*s* ^2^	–1,0	–1,0	0	–2,-1,0	1,2,3	0
_91_Pa	[Rn]5*f* ^2^6*d* ^1^7*s* ^2^	–1,0	–1,0	0	0	2,3,4	3
_92_U	[Rn]5*f* ^3^6*d* ^1^7*s* ^2^	–1,0	–1,0	0	0	2,3,4	3
_93_Np	[Rn]5*f* ^4^6*d* ^1^7*s* ^2^	–1,0	–1,0	0	0	3,4,5	4
_94_Pu	[Rn]5*f* ^6^7*s* ^2^	–1,0	–1,0	–2,-1,0	0	1,2,3	4
_95_Am	[Rn]5*f* ^7^7*s* ^2^	–1,0	–1,0	–2,-1,0	0	1,2,3	4
_96_Cm	[Rn]5*f* ^7^6*d* ^1^7*s* ^2^	–1,0	–1,0	0	0	3,4,5	4
_97_Bk	[Rn]5*f* ^9^7*s* ^2^	–1,0	–1,0	–2,-1,0	0	1,2,3	4
_98_Cf	[Rn]5*f* ^10^7*s* ^2^	–1,0	–1,0	–2,-1,0	0	1,2,3	4
_99_Es	[Rn]5*f* ^11^7*s* ^2^	–1,0	–1,0	–2,-1,0	0	1,2,3	4
_100_Fm	[Rn]5*f* ^12^7*s* ^2^	–1,0	–1,0	–2,-1,0	0	1,2,3	4
_101_Md	[Rn]5*f* ^13^7*s* ^2^	–1,0	–1,0	–2,-1,0	0	1,2,3	4
_102_No	[Rn]5*f* ^14^7*s* ^2^	–1,0	–1,0	–2,-1,0	0	1,2,3	4

### 4.2 aug-RPF-2Z and aug-RPF-3Z Sets

This work also provides versions of the basis sets that are augmented by one extra diffuse function for each symmetry available in the parent RPF-2Z and RPF-3Z sets (*s*, *p*, *d*, *f*, *g*, and *h*). These function exponents are extrapolated by using the p-GCDF parameters and *i* indices one unit smaller than the smallest ones already considered for each symmetry of RPF-2Z and RPF-3Z. These augmented basis sets are named aug-RPF-2Z and aug-RPF-3Z.

## 5 Molecular Calculations

Next, the X2C-SF-CCSD-T calculations of fundamental molecular properties are discussed. These properties include the equilibrium bond lengths (*r*_*e*_), harmonic vibrational frequencies (*ω*_*e*_), and equilibrium dipole moments (*μ*_*e*_). Some experimental data are available for comparison as well.^29–33^ Thus, analyzing the *r*_*e*_, *ω*_*e*_, and *μ*_*e*_ values in Tables 6 [Table tbl7],8, one can see that the results from both triple-*ζ* basis sets, RPF-3Z and dyall.v3z, are close to each other, with maximum absolute errors (MAEs) of only 0.038 Å, 17.6 cm^–1^, and 0.39 D, respectively. However, comparing the values obtained with the experimental data available, RPP-3Z appears to be more accurate than dyall.v3z. Here, it is important to mention that the spin-orbit coupling, which is not considered in X2C-SF calculations, is expected to be of minor relevance for the *r*_*e*_ and *ω*_*e*_ values of closed-shell molecules, although its effect can be more important for dipole moments.^34^ In this context, the mean absolute deviations (MADs) from experiment provided by RPF-3Z are 0.015 Å, 3.9 cm^–1^, and 0.08 D for *r*_*e*_, *ω*_*e*_, and *μ*_*e*_, respectively, while dyall.v3z results in MADs of 0.040 Å, 6.8 cm^–1^, and 0.15 D. These MADs from RPF-3Z are similar to the ones achieved with this same basis set for a group of diatomic molecules constituted by *s*- and *p*-block elements (0.007 Å, 13 cm^–1^, and 0.08 D for *r*_*e*_, *ω*_*e*_, and *μ*_*e*_, respectively).^16^ All these findings support the expected quality of the RPF-3Z set. Of course, the number of molecules investigated here with available experimental results is limited, especially for dipole moments, indicating that future work will be required for a better performance evaluation of these new basis sets.

**Table 6:** Equilibrium Bond Lengths Calculated at the X2C-SF-CCSD-T Level and the Available Experimental Data (in Å)

Molecules	dyall.v2z	dyall.v3z	RPF-2Z	RPF-3Z	Exp^a^
LaF	2.148	2.070	2.043	2.035	2.023
LaCl	2.612	2.546	2.516	2.508	2.498
LaBr	2.763	2.703	2.669	2.667	2.652
LaI	2.995	2.926	2.884	2.898	2.879
CeO	1.750	1.748	1.745	1.751	
CeS	2.246	2.242	2.261	2.236	
PrN	1.773	1.760	1.778	1.759	
AcF	2.246	2.141	2.133	2.126	
AcCl	2.737	2.647	2.627	2.627	
AcBr	2.899	2.813	2.796	2.796	
AcI	3.141	3.049	3.029	3.043	
ThO	1.855	1.849	1.869	1.857	1.840
ThS	2.372	2.357	2.378	2.360	
PaN	1.750	1.740	1.741	1.740	

a Experimental data:^29,30^ .

**Table 7:** Harmonic Vibrational Frequencies Calculated at the X2C-SF-CCSD-T Level and the Available Experimental Data (in cm^–1^)

Molecules^a^	dyall.v2z	dyall.v3z	RPF-2Z	RPF-3Z	Exp^b^
LaF	522.8	561.9	582.1	577.4	574.9
LaCl	329.0	337.4	354.1	345.1	341.4
LaBr	227.8	232.1	240.5	238.0	236.2
LaI	175.2	182.6	191.2	189.3	189.5
CeO	935.2	923.6	950.7	928.0	
CeS	424.7	387.0	463.3	404.6	
PrN	890.7	898.4	874.0	906.0	
AcF	481.1	527.2	546.0	531.8	
AcCl	395.3	305.1	319.9	310.5	
AcBr	192.6	202.2	205.8	203.5	
AcI	146.3	154.5	156.2	156.0	
ThO	874.1	890.0	869.7	884.5	895.8
ThS	484.0	482.4	485.1	484.9	
PaN	1092.6	1117.8	1113.1	1120.8	

a Results were obtained for ^139^LaF, ^139^La^35^Cl, ^139^La^79^Br, ^139^LaI, ^232^Th^16^O, and ^232^Th^32^S.

b Experimental data:^29,30^ .

**Table 8:** Equilibrium Dipole Moments Calculated at the X2C-SF-CCSD-T Level and the Available Experimental Data (in Debye)

Molecules	dyall.v2z	dyall.v3z	RPF-2Z	RPF-3Z	Exp^a^
LaF	2.720	2.130	1.509	1.737	1.808
LaCl	3.528	3.467	3.104	3.184	
LaBr	3.531	3.705	3.421	3.523	
LaI	3.746	4.031	3.692	4.017	
CeO	0.341	0.539	0.428	0.610	
CeS	2.497	2.807	3.082	2.702	
PrN	7.159	7.327	7.592	7.339	
AcF	3.395	2.432	2.346	2.291	
AcCl	4.105	3.611	3.433	3.480	
AcBr	4.130	3.831	3.690	3.747	
AcI	4.302	4.073	3.846	4.073	
ThO	2.673	2.742	2.886	2.850	2.782
ThS	4.181	4.496	4.477	4.477	4.58
PaN	2.908	2.961	2.865	2.959	

a Experimental data:^31–33^ .

The MAEs obtained between the double-*ζ* sets (RPF-2Z and dyall.v2z) for *r*_*e*_, *ω*_*e*_, and *μ*_*e*_ are larger than the ones previously discussed: 0.113 Å, 75.4 cm^–1^, and 1.21 D, respectively. Again, by using the experimental data as reference, RPF-2Z (MADs of 0.018 Å, 10.4 cm^–1^, and 0.17 D) seems to be more accurate than dyall.v2z (MADs of 0.096 Å, 21.8 cm^–1^, and 0.47 D). The MADs from the RPF-2Z set are again similar to the ones obtained with *s*- and *p*-block element compounds (0.020 Å, 32 cm^–1^, and 0.10 D for *r*_*e*_, *ω*_*e*_, and *μ*_*e*_, respectively).^16^ Hence, the performance from RPF-2Z is in accordance with the quality expected from a double-*ζ* basis set.

Finally, it is important to reinforce that the results from the dyall.v2z and dyall.v3z sets for these molecules are in much larger discrepancy with the experimental data of *r*_e_ than is normally observed for diatomic systems of *s*- and *p*-block elements.^16^ This outcome might be caused by an insufficient number of C/P functions for lanthanum, since significant deviations in such property results are now observed from calculations done with those popular sets for the lanthanum halides investigated here.

## 6 Conclusions

New relativistic prolapse-free Gaussian basis sets of double- and triple-*ζ* quality (RPF-2Z and RPF-3Z) are provided by combining selected C/P functions addressing the valence space with primitives previously developed for the ground-state electron configuration of *f*-block elements (SRPF and MRPF). Moreover, augmented versions of these sets, containing extra diffuse functions for every symmetry available are also presented: aug-RPF-2Z and aug-RPF-3Z. In this context, the p-GCDF method is used for achieving the exponents of the C/P functions. The expected quality of these new basis sets is clearly demonstrated in comparative molecular property investigations including other popular basis sets of similar quality, such as dyall.v2z and dyall.v3z.

Finally, since these new basis sets are designed to be free of variational prolapse, they are also recommended for the treatment of far more challenging properties that depend strongly on the innermost electron densities.

## Supplementary Material









## Footnotes

1 The correct SRPF size for this configuration of Gd in Table 6 of Ref. 11 is 25*s*18*p*13*d*9*f*.

## Notes

Relativistic prolapse-free Gaussian basis sets of double-zeta quality: RPF-2Z (TXT)
Relativistic prolapse-free Gaussian basis sets of double-zeta quality: RPF-3Z (TXT)
Relativistic prolapse-free Gaussian basis sets of double-zeta quality: aug-RPF-2Z (TXT)
Relativistic prolapse-free Gaussian basis sets of double-zeta quality: aug-RPF-3Z (TXT)
